# Placental endocrine function is controlled by maternal gut *Bifidobacterium* in germ-free mice

**DOI:** 10.1186/s12967-025-07198-4

**Published:** 2025-10-07

**Authors:** Jorge Lopez-Tello, Raymond Kiu, Zoe Schofield, Matthew J. Dalby, Douwe van Sinderen, Gwénaëlle Le Gall, Lindsay J. Hall, Amanda N. Sferruzzi-Perri

**Affiliations:** 1 Department of Physiology, Development, and Neuroscience, The Loke Centre for Trophoblast Research, University of Cambridge, Cambridge, UK; 2 Department of Physiology, Faculty of Medicine, Autonomous University of Madrid, Madrid, Spain; 3 Food, Microbiome and Health, Quadram Institute Bioscience, Norwich Research Park, Norwich, UK; 4 APC Microbiome Institute, University College Cork, Cork, Ireland; 5 Norwich Medical School, University of East Anglia, Bob Champion Research, and Education Building, James Watson Road, Norwich Research Park, Norwich, NR4 7UQ UK; 6 Department of Microbes, Infection, and Microbiomes, School of Infection, Inflammation and Immunology, College of Medicine and Health, University of Birmingham, Birmingham, UK; 7 Institute of Microbiology & Infection, University of Birmingham, Birmingham, UK

**Keywords:** Placenta, Microbiota, Endocrinology, Bifidobacterium, Gut, Pregnancy

## Abstract

**Background:**

Recent studies have shown that the maternal gut microbiota can regulate placental growth, particularly the transport region, in association with fetal growth. However, the specific role of certain microorganisms in modulating the hormonal production of the placenta, which is critical for supporting fetal development and maintaining a healthy pregnancy, remains largely unexplored. In this context, the objective of this study is to determine whether the maternal colonisation with the early life gut bacterium *Bifidobacterium breve* UCC2003 regulates placental endocrine function.

**Methods:**

Pregnant germ-free mice were colonized with or without *Bifidobacterium breve* UCC2003 (BIF) during pregnancy. The endocrine region of the placenta (junctional zone, Jz) was collected to assess its metabolic profile using metabolomics, the expression of key nutrient uptake genes, hormones and synthetic genes by qPCR, and proteome using LC-MS/MS.

**Results:**

BIF colonised dams had increased lactate and taurine concentrations in the placental Jz. BIF presence was also associated with upregulated expression of nutrient carriers, particularly those involved in large neutral amino acid and monocarboxylate uptake (e.g., *Slc7a8* and *Slc16a4*). Additionally, key hormones, such as prolactins and pregnancy-specific glycoproteins, were upregulated. The Jz proteome was changed in BIF colonised dams, with over 400 proteins dysregulated. Pathway analysis revealed more than 150 biological processes were altered, including transcriptional activity, protein synthesis, cell cycle progression, and metabolic regulation. Proteins regulated by BIF in the placental Jz were correlated with fetal growth and nutrient levels (namely glucose). Notably, maternal-associated BIF reduced the number of fetal resorptions (early fetal loss).

**Conclusions:**

In germ-free mice, maternal-associated gut *Bifidobacterium breve* UCC2003 regulates placental endocrine capacity, by altering its metabolic profile and ability to produce endocrine factors. This study provides the first clear evidence that the maternal gut microbiota not only influences placental transport function, but also regulates its endocrine outputs.

**Graphical Abstract:**

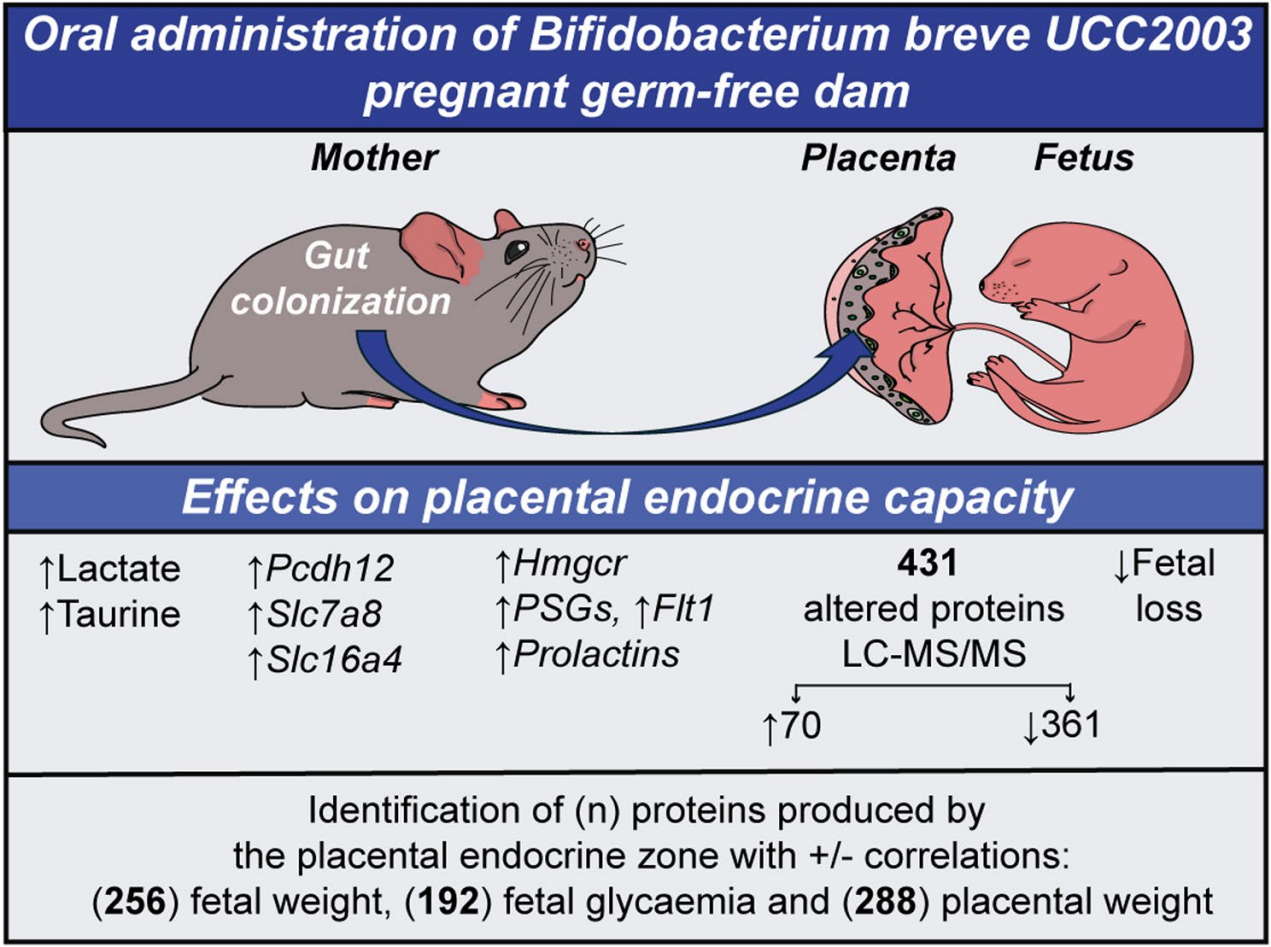

**Supplementary Information:**

The online version contains supplementary material available at 10.1186/s12967-025-07198-4.

## Background

An individual’s metabolism is strongly influenced by the composition of their gut microbiota [1–3], which in turn plays a major role in digesting food, extracting nutrients, regulating metabolism, and influencing immune system development. Through fermentation of complex carbohydrates, the microbiota produces short chain fatty acids (SCFAs) such as acetate, propionate, or butyrate [4], as well as other metabolites like lactate, a common short chain hydroxy–fatty acid. These microbial metabolites have been shown to be involved in various physiological functions, including regulating epithelial barrier function, mucosal and systemic immunity [5]. Moreover, SCFAs act not only locally in the gut but also circulate in the bloodstream to reach other organs such as the brain, adipose tissue and liver [3, 5, 6]. For example, SCFAs produced by the gut microbiota can modulate the secretion of certain neurotransmitters, such as serotonin, and can also influence the blood–brain barrier and vagus nerve activity [7, 8]. Thereby, supporting the concept of communication between the gut microbiota and host organ axes, including the gut–brain axis [8, 9].

The gut microbiota plays a crucial role in pregnancy, as maternal metabolism must adapt to optimise nutrient absorption and storage while ensuring adequate resources are allocated for fetal growth [10, 11]. During pregnancy, the composition of the maternal gut microbiota undergoes significant changes [18, 19], with comparisons between the first and third trimesters revealing striking differences [19]. Among the bacterial genera influenced by pregnancy, *Bifidobacterium* - a commensal genus with pro-homeostatic and anti-inflammatory immunomodulatory properties [20] - increases in late pregnancy in both humans and mice [19]. This expansion is partly driven by pregnancy-related hormones such as progesterone, which promote *Bifidobacterium* abundance [19]. Several reports have found that alterations in *Bifidobacterium* levels are linked to pregnancy complications, including preterm birth [12] and preeclampsia [13], while broader microbiome perturbations are associated with conditions like gestational diabetes [14–16] and fetal growth restriction [17, 18]. Moreover, we and others have found that administering specific *Bifidobacterium* strains to pregnant mice improves fetal growth and glycaemia, and induces beneficial changes in the transport region of the placenta [19–22]. However, to our knowledge, the concept of bacteria in the maternal gut remotely influencing the endocrine function of the placenta remains unexplored.

The placenta is the vital interface between the mother and fetus, serving as the lifeline for fetal growth and development. This transient organ facilitates transport of oxygen and essential nutrients, whilst also protecting the fetus from immune rejection by the mother [23–25]. Beyond these fundamental roles, the placenta functions as a major endocrine organ, secreting a wide range of hormones crucial for maternal support of pregnancy [23, 26–28]. However, because placental growth depends principally on maternal resources, placental structure and function are heavily regulated by maternal nutritional and metabolic status [29–33]. Studies in humans and animal models have demonstrated that disruptions in maternal diet or metabolism can impair placental development and compromise fetal growth [30, 31, 33, 34]. Consequently, alterations in the maternal gut microbiota, an emerging regulator of host metabolism, may also shape placental physiology and impact pregnancy outcomes.

Here, we employ our previously validated germ-free (GF) model [19, 20] to investigate how the maternal gut bacterium *Bifidobacterium breve* UCC2003 (*B. breve*) influences placental endocrine function. Our new results further demonstrate that a single gut bacterium can modulate placental endocrine function. Specifically, we observed changes in the abundance of over 400 proteins involved in a range of biological processes, including metabolism, translation, and cell cycle regulation. These findings were related to improved fetal growth and viability. They also provide novel insights into how the gut microbiota can regulate placental function, opening new avenues for novel therapeutic strategies aimed at optimizing pregnancy outcomes.

## Methods

### Mouse model and administration of probiotic

**Fig. 1:**
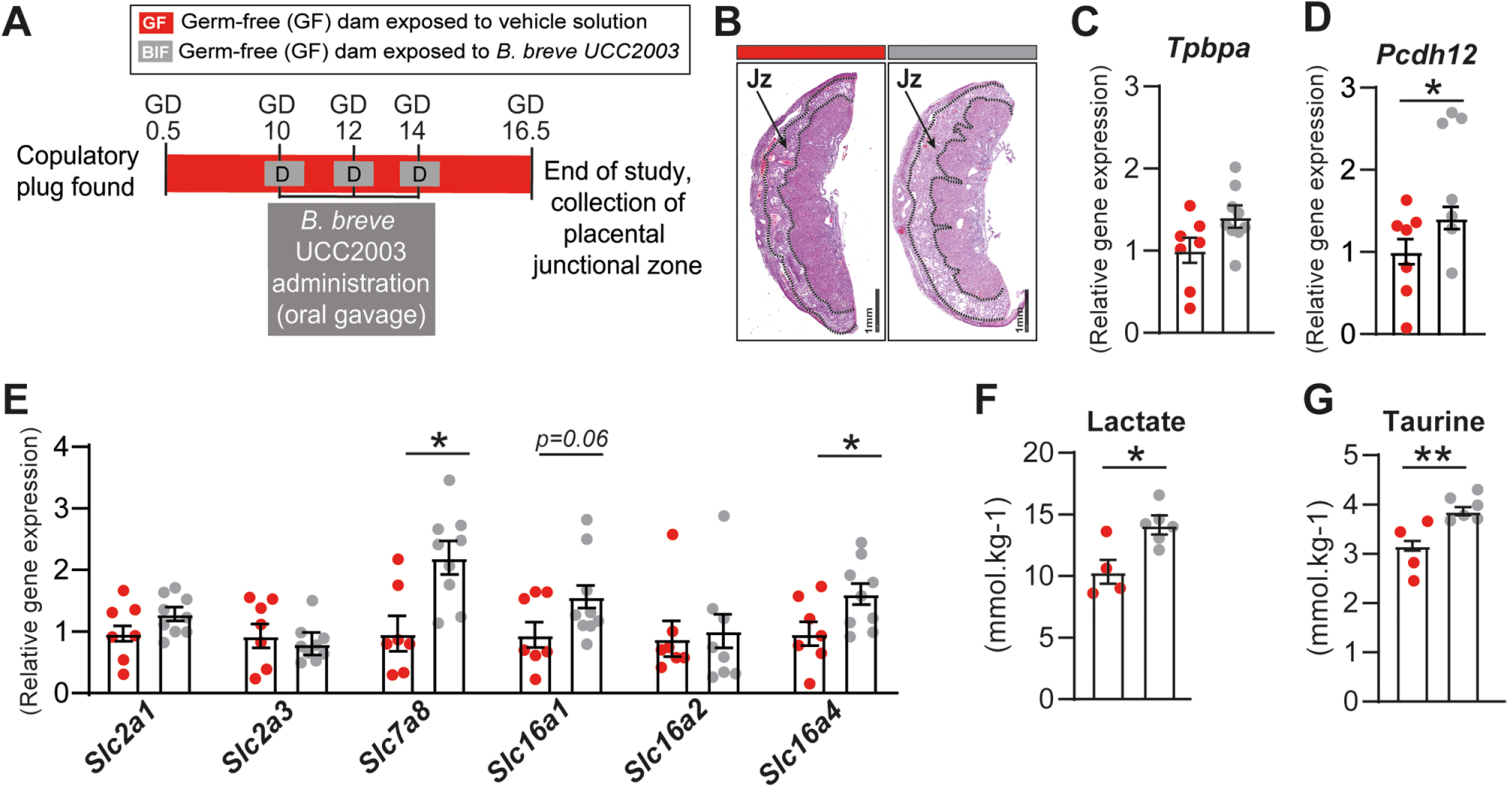
Maternal *B. breve* supplementation induces changes in specific metabolites and nutrient carriers in the endocrine zone of the mouse placenta. **(A)** Experimental design (GD: gestational day). **(B)** Representative images of placentas with the endocrine region (junctional zone, Jz) delineated. Scale bar 1 mm. **(C-E)** Relative mRNA levels of *Tpbpa*,* Pcdh12* and nutrient carrier proteins in the Jz for BIF compared to GF mice determined by qPCR. **(F-G)** Concentrations of lactate and taurine in the Jz determined by metabolomics using nuclear magnetic resonance (NMR) spectroscopy. Data are presented means ± SEM, with individual datapoints shown. *P < 0.05; **P < 0.01

The experimental design (Fig. 1A) and husbandry conditions were described in detail in our previous publications [19, 20]. All details regarding the bacterial strain, preparation, dosing and efficiency of *Bifidobacterium breve* UCC2003 maternal gut colonization can be also found in our previous publication [19].

All animal work was approved by the UK Home Office and the University of East Anglia Ethical Review Committee under project license PDADA1B0C. Briefly, female germ-free (GF) C57BL/6J mice were time-mated, and on gestational day (GD) 10, six pregnant GF mice received, via oral gavage, 100 µL of reconstituted lyophilized *Bifidobacterium breve* UCC2003 (BIF group). Another cohort of five GF dams received the vehicle solution (GF group). To ensure colonization of the maternal digestive tract, two additional doses were administered on GD12 and GD14. Colony-forming unit (CFU) data confirming the levels and persistence of *Bifidobacterium breve* colonization throughout the study period are available in our previous publication [19] and in Figure S1. On GD16.5, all pregnant dams were sacrificed by cervical dislocation, the gravid uterus was retrieved, and the number of resorptions and viable conceptuses was recorded. This time point of tissue collection was selected because it corresponds to the period of maximal placental growth [35], suggesting that placental endocrine output may be most extensive at this stage [26]. Moreover, this GD coincides with the stage when the gravid mouse exhibits peak glucose intolerance and insulin resistance [36].

Placentas were collected, rinsed in PBS, and halved for morphological analysis or mechanically separated under a microscope into enriched endocrine tissue (junctional zone, Jz) and vascular/transport tissue (labyrinth zone, Lz) for molecular analysis. The separated portions of the placental Jz were either (1) snap-frozen in liquid nitrogen and stored in Eppendorf tubes for gene expression and metabolic analyses or (2) preserved in cryopreservation liquid (0.21 M mannitol, 0.07 M sucrose, 30% DMSO in H₂O, pH 7.5) for proteomic analysis.

### Placental sex determination

Selected placental Jz samples were sexed by detecting the *Sry* gene [37]. Briefly, samples were incubated overnight at 55 °C in lysis buffer containing KCl, 1 M Tris-HCl, 1 M MgCl_2_, gelatin, Tween-20, Nonidet P-40, and proteinase K. The following day, the samples were heated at 94 °C for a total of 15 min. Then, samples were combined with the Taq Ready PCR system (Sigma-Aldrich) and specific primers for *Sry* (Sry: FPrimer: 5′-GTGGGTTCCTGTCCCACTGC-3′, RPrimer: 5′-GGCCATGTCAAGCGCCCCAT-3′) and PCR autosomal gene control (FPrimer: 5′-TGGTTGGCATTTTATCCCTAGAAC-3′, RPrimer: 5′-GCAACATGGCAACTGGAAACA-3′). PCR products were assessed by agarose gel electrophoresis. Distribution of sex for each of the techniques used can be found in Table S1, confirming that each analysis was fetal sex-balanced.

### Placental RNA extraction and gene expression analysis

RNA extraction and qPCR analysis were conducted on placental Jz samples as described previously [26]. Gene expression was normalized to the geometric mean of two reference genes, *Actb* and *Krt18*, which remained stably expressed across the groups. Relative gene expression levels were calculated using the 2-ΔΔCt method and the list of primers used for the analysis is provided in Table S2.

### Metabolomic analysis

Metabolomic analysis of placental Jz tissue was conducted as described in our previous study [19].

### Proteomic analysis, sample dissolution, TMT labelling and Reverse-Phase fractionation for proteomic analysis

Placental Jz tissues were resuspended in lysis buffer containing 100mM Triethylammonium bicarbonate (TEAB, Sigma), 10% isopropanol, 50mM NaCl, 1% SDC (Sodium Deoxy Cholate) with 1X Protease and Phosphatase inhibitors and Nuclease. Samples were incubated at room temperature (RT) for 15 min, followed by Percelly’s with 2 × 30 s Cycle of 6,800 rpm with 30s pause. Protein concentration was estimated using Bradford assay according to manufacturer’s instructions (BIO-RAD-Quick start). 50ug of total protein was taken in a total volume of 20 µl across all the samples using 0.1 M TEAB. Reduced with 2ul of 50mM tris-2-caraboxymethyl phosphine (TCEP, Sigma) along with alkylation with 1ul of 200mM Iodoacetamide (IAA, Sigma) for 1 h at RT. Then protein samples were digested overnight at 37 °C using trypsin solution at ratio protein/trypsin ~ 1:30. The next day, protein digest was labelled with the TMTpro-18plex reagents (Thermo Scientific) for 1 h. The reaction was quenched with 8 µL of 5% hydroxylamine (Thermo Scientific) for 15 min at RT. All the samples were mixed and the SDC removed using a final concentration of 5% formic acid. Later the pool was dried using speed vac concentrator. The dried TMT mix was fractionated on a Dionex Ultimate 3000 system at high pH using the X-Bridge C18 column (3.5 μm, 2.1 × 150 mm, Waters) with 90 min linear gradient from 5% to 95% acetonitrile contained 20mM ammonium hydroxide at a flow rate of 0.2 ml/min. Peptides fractions were collected between 20 and 55 min and were dried with speed vacuum concentrator. Each fraction was reconstituted in 0.1% formic acid for liquid chromatography tandem mass spectrometry (LC–MS/MS) analysis.

### Liquid chromatography–mass spectrometry (LC-MS/MS)

Peptide fractions were analysed on a Dionex Ultimate 3000 system coupled with the nano-ESI source Fusion Lumos Orbitrap Mass Spectrometer (Thermo Scientific). Peptides were trapped on a 100 μm ID X 2 cm microcapillary C18 column (5 μm, 100 A) followed by 2 h elution using 75 μm ID X 50 cm C18 RP column (3 μm, 100 A) at 300nl/min flow rate. In each data collection cycle, one full MS scan (380–1,500 m/z) was acquired in the Orbitrap (120 K resolution, Normalized automatic gain control (AGC) target of 75% and Maximum Injection Time (MIT) of 100 ms). The subsequent MS2 was conducted with a top speed approach using a 3-s duration. The most abundant ions were selected for fragmentation by collision induced dissociation (CID). CID was performed with a fixed collision energy of 32%, a Normalized AGC target of 100%, an isolation window of 0.7 Da, a MIT of 50 ms. Previously analysed precursor ions were dynamically excluded for 45s. During the MS3 analysis for TMT quantification, precursor ion selection was based on the previous MS2 scan and isolated using a 2.0Da m/z window. MS2–MS3 was conducted using sequential precursor selection (SPS) methodology with the top10 settings. HCD was used for the MS3, with 50% collision energy and reporter ions were detected using the Orbitrap (50 K resolution, a Normalized AGC target of 200% and MIT of 105 ms).

### Statistical analysis

Statistical analysis was performed with GraphPad Prism software, SAS/STAT 9.0, and R. Gene expression analysis was analysed by one-way ANOVA, with the group as fixed effect and each fetus treated as a repeated measure. Fisher’s test was employed for means comparisons (linear mixed model - MIXED model), with litter size as a covariate. The number of resorptions per litter was analysed by Student’s *t*-test. Metabolomic analysis was also performed using one-way ANOVA, with the group as a fixed effect, and means comparisons were made by Fisher’s test (general linear model-GLM model, with one fetus selected per litter). Statistical significance was set at *p* < 0.05. Correlation analysis was performed by Spearman’s analysis followed by false discovery rate adjustment.

For LC-MS/MS data processing, Proteome Discoverer 2.4. (Thermo Scientific) was used to process CID tandem mass spectra. The SequestHT search engine was applied, and all spectra were searched against the Uniprot Homo sapiens FASTA database (taxon ID 9606 - Version June 2023). All searches were performed using static modification TMTpro (+ 304.207 Da) at any N-terminus/lysines and carbamidomethyl at cysteines (+ 57.021 Da). Methionine oxidation (+ 15.9949 Da) and deamidation on asparagine and glutamine (+ 0.984) were included as dynamic modifications. Mass spectra were searched using precursor ion tolerance of 20 ppm and fragment ion tolerance 0.5 Da. For peptide confidence, 1% FDR was applied and peptides uniquely matched to a protein were used for quantification. Figures in the manuscript are displayed as mean ± SEM alongside individual data points (raw data) shown.

## Results

### Maternal gut B. breve UCC2003 enhances nutrient carrier expression and metabolite levels in the endocrine zone of the mouse placenta

In our previous study we found that *B. breve* treatment of GF mice (experimental design shown in Fig. 1A) did not affect the size of the placental endocrine zone (Jz) [19]. Consistent with this, the mRNA levels of *Tpbpa*, a gene specifically expressed in the Jz, remained unchanged in the placenta following BIF treatment [38] (Fig. 1B-C). However, the expression of *Pcdh12*, an important trophoblast glycogen cell marker [39], was increased in the BIF group (Fig. 1D). Moreover, considering that previous studies have shown that the endocrine function of the mouse placenta is strongly influenced by imprinting genes, particularly *Igf2*, *H19* and *Peg3* [26, 40–42], the mRNA levels of these imprinted genes were examined. Expression of paternally-expressed (*Igf2* and *Peg3*) and maternally-expressed (*H19*) genes was shown to be unaltered in the Jz in response to BIF treatment (Figure S2).

We then analysed the expression of *Slc7a8* and *Slc16a4*, which facilitate in the uptake of large neutral amino acids and monocarboxylates such as lactate, and were shown to be significantly increased in the BIF versus control GF group (Fig. 1E). In contrast, no changes were observed in mRNA levels of glucose carriers *Slc2a1* and *Slc2a3*, or in the expression of other monocarboxylates transporters, namely *Slc16a1* and *Slc16a2* (Fig. 1E). Additionally, metabolomic analysis of the Jz revealed increased concentrations of lactate and taurine in the BIF-treated group (Fig. 1F-G). Further details about the additional metabolites measured can be found in Table S3. Taking together, these data suggest that maternal gut *B. breve* can exert metabolic changes in the endocrine layer of the mouse placenta.

### Maternal gut B. breve UCC2003 reduces the appearance of resorption and upregulates placental steroidogenesis and pregnancy specific-hormones

**Fig. 2:**
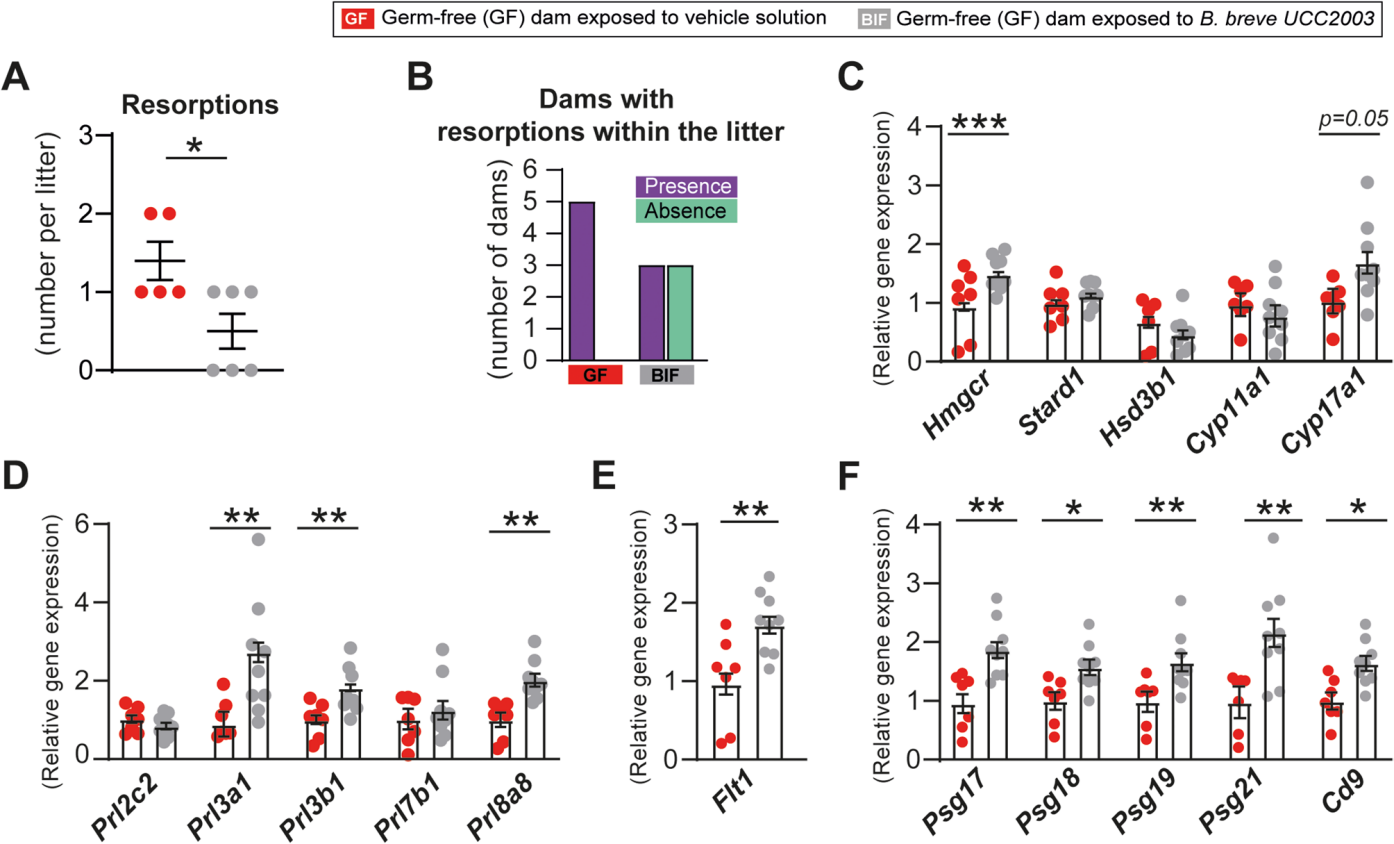
Maternal gut *B. breve* increases steroidogenic enzyme and hormone abundance, including prolactins and pregnancy-specific glycoproteins, and reducing the incidence of fetal resorptions in mice. **(A-B)** Number of resorptions per litter and number of dams with resorptions. **(C)** Relative mRNA levels of enzymes involved in steroidogenesis **(D)**, prolactins **(E)**, *Flt1*, **(F)** pregnancy-specific glycoproteins (PSGs) and proposed PSG receptor *Cd9* determined by qPCR. Data are presented as means ± SEM, with individual datapoints shown. *P < 0.05; **P < 0.01; ***P < 0.001

In our previous publication we reported that BIF treatment did not affect the number of viable fetuses on day 16 of gestation (similar viable litter size number) [19]. However, we found that the incidence of fetal resorptions/loss in GF mice was reduced by the BIF treatment (Fig. 2A). Notably, the number of pregnant mice with resorptions within their litter was halved by BIF supplementation (Fig. 2B).

Considering that fetal loss is influenced by placental hormone production [43], we analyzed enzymes and hormones previously implicated in steroidogenesis and fetal growth regulation, including *Cyp17a1*, *Prl3a1*, and *Psg21* [44] in BIF-treated GF pregnant mice. Among the steroidogenic enzymes measured, *Hmgcr* expression was significantly increased in the placenta following BIF treatment (Fig. 2C). Additionally, *Cyp17a1* showed a trend toward higher expression (*p* = 0.05). Other enzymes, including *Stard1*, *Hsd3b1*, and *Cyp11a1*, remained unchanged (Fig. 2C).

We also quantified the mRNA levels of five prolactin variants (*Prl2c2*, *Prl3a1*, *Prl3b1*, *Prl7b1*, and *Prl8a8*), four pregnancy-specific glycoproteins (PSGs: *Psg17*, *Psg18*, *Psg19*, *Psg21*), the putative PSG receptor (*Cd9*), as well as *Flt1* (FMS-like tyrosine kinase 1), a key regulator of placental angiogenesis [45]. This revealed that BIF supplementation of GF mice transcriptionally upregulates *Prl3a1*, *Prl3b1*, *Prl8a8*, *Flt1*, and all measured PSGs, and *Cd9* (Fig. 2D-F).

Collectively, these results demonstrate, that a maternal gut bacterium can remotely regulate the mRNA levels of key proteins involved in the endocrine function of the mouse placenta with consequences for fetal survival.

### Maternal gut B. breve UCC2003 alters the placenta proteome, affecting 400 + proteins involved in transcription, translation, and metabolism

**Fig. 3:**
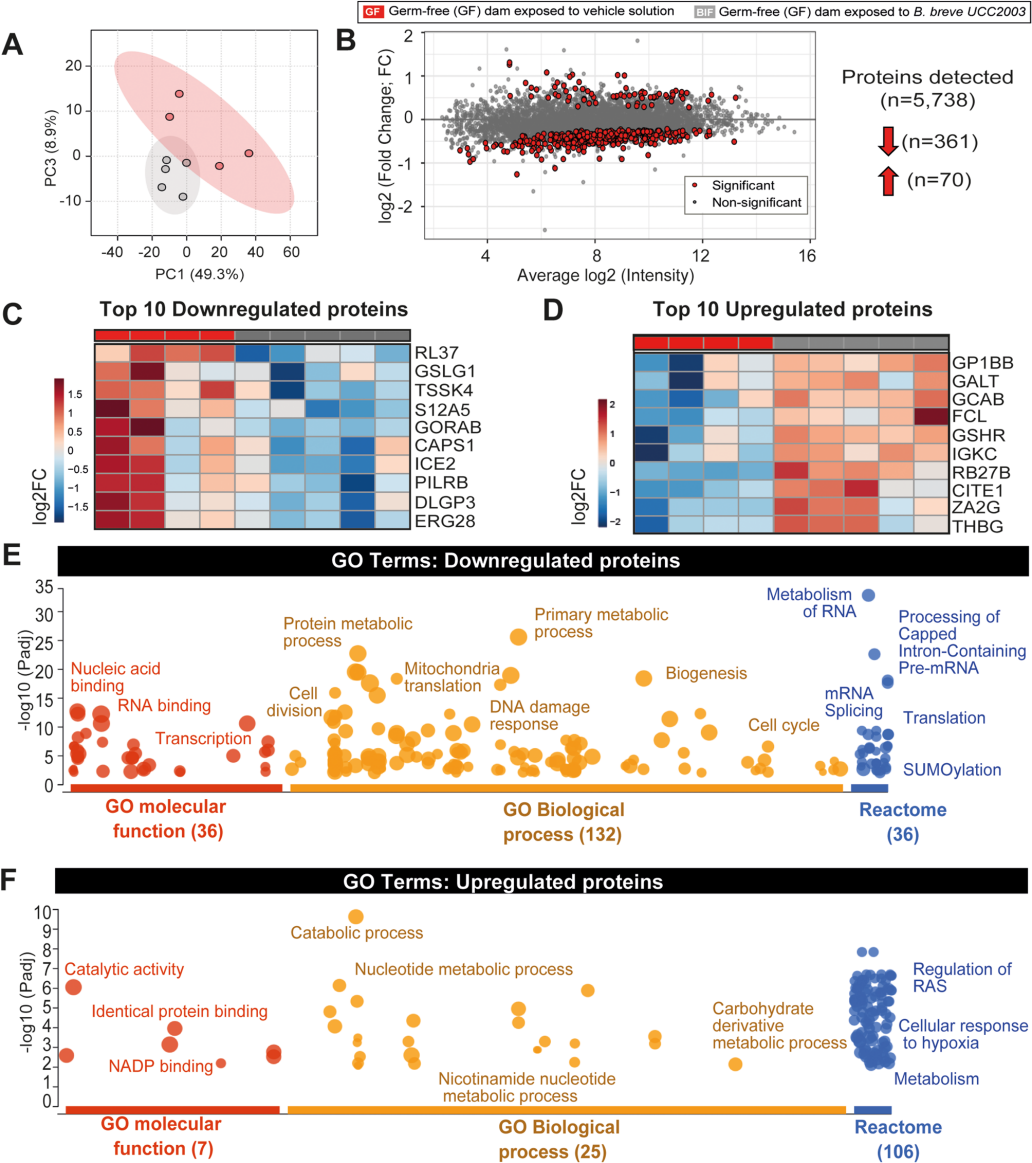
Maternal gut *B. breve* alters the endocrine capacity of the mouse placenta. **(A)** PCA plot showing distinct clustering between GF (grey) and BIF (red) groups. Proteomics analysis conducted by LC-MS/MS in microdissected placental junctional zone tissue. **(B)** Bland-Altman plot displaying log2 fold changes in protein abundance *versus* average log2 intensity. Note that red dots are significantly altered proteins. **(C-D)** Heatmap showing the top 10 most downregulated and upregulated proteins identified. **(E-F)** Gene Ontology (GO) enrichment analysis for downregulated and upregulated terms. Further details on the dysregulated proteins and GO terms are available in Table S4. The enrichment analysis was conducted exclusively on the list of dysregulated proteins

To comprehensively assess how maternal BIF could influence placental endocrine capacity, global proteomic analysis of mouse Jz samples was performed using LC-MS/MS. Principal Component Analysis revealed a clear separation of the two experimental groups, indicating significant alterations in the placental proteome following BIF administration of GF mice during pregnancy (Fig. 3A).

LC-MS/MS analysis identified over 5,000 proteins, of which 431 were significantly differentially expressed: 361 were downregulated and 70 were upregulated following BIF administration (Fig. 3B–D). We next investigated whether the mRNA-level changes previously observed for hormone-related and other key genes (Figs. 1 and 2) were reflected at the protein level. Interestingly, while PCDH12 was upregulated at the mRNA level, its protein expression remained unchanged. In contrast, PEG3 showed a significant decrease in protein levels in the BIF-treated group, despite no apparent change in its transcript levels. Protein expression of IGF2 remained consistent between groups (Table S4).

Among nutrient transporters, the protein level of SLC7A8, which was upregulated at the mRNA level, remained unchanged. Another transporter, SLC16A4, also showed increased mRNA expression but was not detected at the protein level. Interestingly, SLC16A1, which exhibited a trend toward increased mRNA expression, was significantly downregulated at the protein level in the BIF group (Table S4).

LC-MS/MS detected 3 out of the 5 isoforms of prolactin previously identified at the mRNA level. Notably, PRL3B1 and PRL8A8, which were upregulated at the transcript level, showed no corresponding increase in protein abundance (Table S4). The other detected isoform of prolactin, PRL7B1 was also unaltered between groups. Similarly, protein levels of FLT1 and CD9 were unchanged. Additionally, none of the four PSGs assessed were detected by LC-MS/MS. Taken together, these data suggest a disconnect between mRNA expression and protein production for several genes, highlighting potential post-transcriptional regulatory mechanisms or limitations in protein detection by LC-MS/MS [46, 47].

Functional enrichment analysis of the 431 differentially expressed proteins detected by LC-MS/MS (GO, Gene Ontology – Table S4) identified a total of 47 altered molecular function terms, including 36 downregulated terms such as nucleic acid binding (e.g., SF3A1, FXR1, HDAC1) and transcription (e.g., FXR1, ASCC3, EIF3C). Additionally, 7 terms were upregulated, including NADP binding (e.g., GSR, ME1, G6PDX), catalytic activity (e.g., GALT, GFUS, GSR), and protein binding (e.g., ALDOA, TXNRD1, ALDH9A1) (Table S4).

Analysis of GO biological processes identified 157 terms altered by BIF, with 132 downregulated and 25 upregulated (Fig. 3E). Downregulated biological processes primarily involved transcription, protein synthesis, cell cycle progression, and metabolic regulation (Fig. 3E and Table S4). For instance, TSSK4 and ICE2, two of the most downregulated proteins (Fig. 3C), are implicated in cell cycle regulation, protein/RNA metabolic processes, and transcriptional activity. GORAB, another highly downregulated protein, is involved in organelle organization and cellular component biogenesis.

In contrast, upregulated processes of the 431 differentially expressed proteins suggest an overall increase in metabolic activity, particularly in nucleotide, small molecule, and protein catabolism. Proteins such as GALT, PSMA1, and PSMB1, which are critical to these pathways, were significantly increased. Notably, GALT, which also participates in carbohydrate and nucleoside phosphate metabolism, was among the top 10 most upregulated proteins in response to BIF administration (Fig. 3D).

Using the list of dysregulated proteins, REACTOME pathway analysis identified similar pathways to those previously described, with a total of 142 biological pathways altered. Of the 36 downregulated pathways, many were involved in RNA processing, transcription, translation, and DNA repair (Fig. 3E and Table S4). Meanwhile, among the 106 upregulated pathways, BIF modulated key signalling pathways, including noncanonical NF-kB signalling, (Dectin-1 mediated NF-kB activation and TNFR2 non-canonical NF-kB pathway), cell cycle regulation (e.g., G1/S DNA damage checkpoints and p53-independent G1/S DNA damage checkpoint), stress responses (e.g., MAPK signalling and the RAF/MAP kinase cascade), and metabolism (e.g., polyamines, amino acid and nucleotide metabolism) (Fig. 3F and Table S4).

Collectively, these data offer new insights into how maternal gut *B. breve* UCC2003 regulates placental function. Our comprehensive proteomic analysis of endocrine-enriched placental tissue reveals significant alterations in key biological processes. These findings underscore the potential of maternal gut microbiota, particularly *B. breve*, in modulating placental endocrine function and highlight broader implications for understanding the impact of the microbiome on maternal and fetal health.

### Identification of novel placental proteins involved in the regulation of feto-placental growth and metabolism

**Fig. 4:**
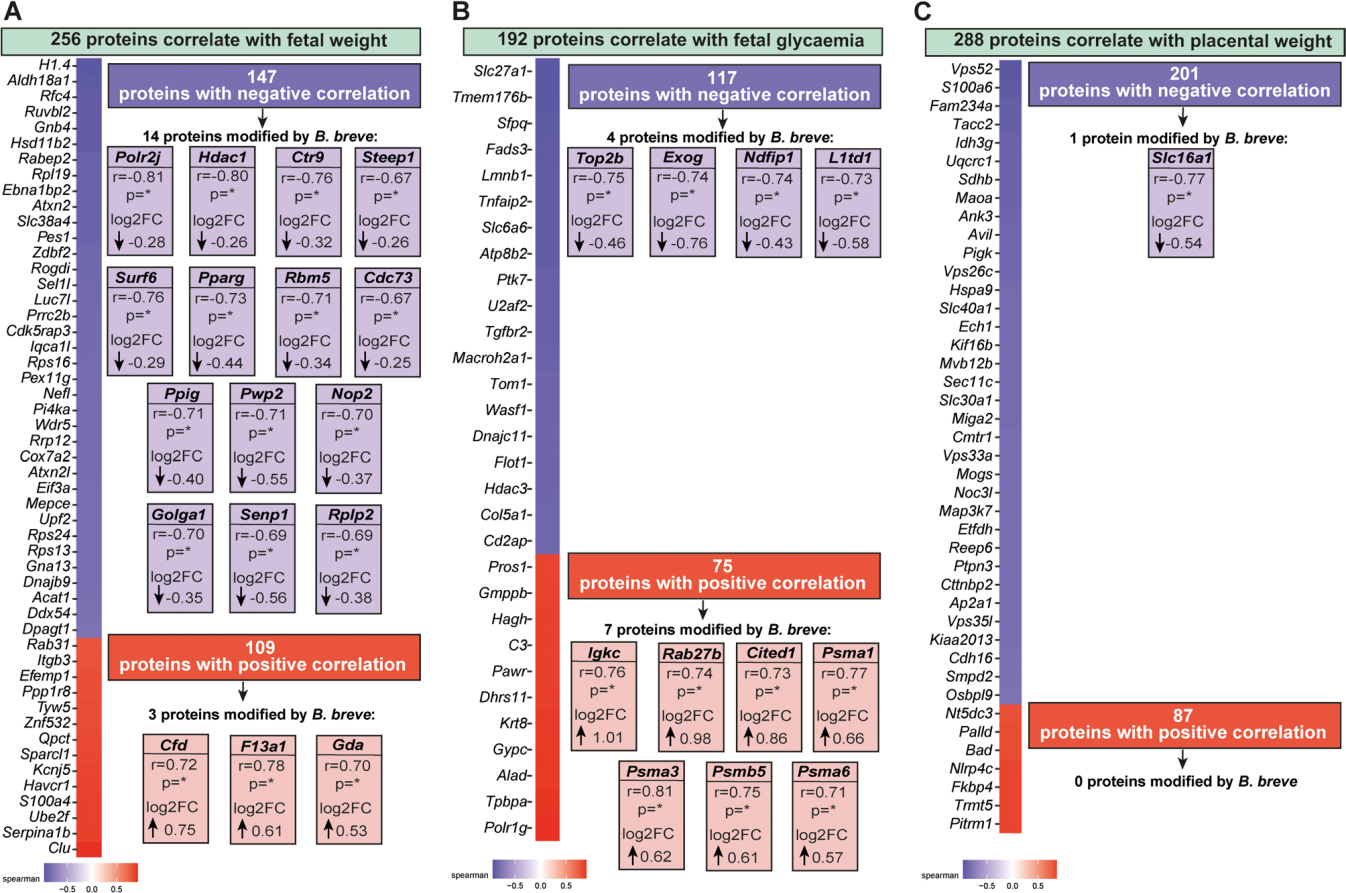
Correlation analysis between all the placental proteins detected in the junctional zone with feto-placental weight and glycemia. **(A)** Proteins correlating with fetal weight (*n* = 256, 147 with negatively and 109 positively correlated). **(B)** Proteins correlating with fetal glycaemia (*n* = 192, 117 with negatively and 75 positively correlated). **(C)** Proteins correlating with placental weight (*n* = 288, 166 with negatively and 122 positively correlated). Spearman’s correlation coefficients (r) and statistical significance (p) for proteins also regulated by maternal gut *B. breve*. Log2 fold change (log2FC) values indicate the direction and magnitude of protein expression changes in response to *B. breve* administration. Proteins with negative correlation are displayed in purple, whist those with positive correlation are displayed in red. Due to space limitations, only the most relevant proteins are shown, and their identifiers are presented using gene names for clarity and conciseness. For further details, refer to Supplementary Table 4.

To uncover new placental proteins involved in the regulation of feto-placental growth, a correlation analysis was performed using the complete list of proteins (5,738 proteins) detected by LC-MS/MS. Protein levels were correlated with fetal weight, fetal glycaemia, and placental weight (Table S4 and Fig. 4).

The analysis identified 256 placental proteins associated with fetal weight (Fig. 4A), including 147 negatively correlated and 109 positively correlated proteins. Notably, among the negatively correlated proteins, 14 were downregulated by BIF, including PPARG and SENP1. Conversely, among the positively correlated proteins, BIF administration was associated with the upregulation of three proteins: CFD, F13A1, and GDA.

For placental proteins correlated with fetal glucose levels (Fig. 4B), 117 were negative correlated, including four that were downregulated by BIF treatment (TOP2B, EXOG, NDFIP1, and L1TD1). Among the 75 positively correlated proteins, seven were modulated by BIF, including CITED1, IGKC, and PSMA1.

Regarding placental weight (Fig. 4C), 288 placental proteins showed a significant correlation (201 negatively and 87 positively correlated). However, only one protein, SLC16A1, was modulated following BIF treatment of pregnant GF mice.

Collectively, these findings reveal novel placental proteins involved in the regulation of fetoplacental growth and fetal glycaemia, highlighting promising targets for future investigation. They also reinforce the emerging role of maternal gut bacteria in modulating fetoplacental development.

## Discussion

In recent years, numerous studies have explored the interplay between the gut microbiota and other host organs, including the brain, liver, and adipose tissue [48–51]. However, despite ongoing research, our understanding of how the maternal gut microbiota shapes placental growth and function remains limited, even though the placenta plays a pivotal role in fetal development and maternal health.

Building on our previous study, which demonstrated that the maternal microbiota regulates placental labyrinth zone structure and the abundance of key nutrient transporters with consequences for fetal growth and metabolism [19, 20], here we have uncovered a new role played by *B. breve.* Specifically, our study indicates that a single maternal gut bacterium, *B. breve*, can control the endocrine function of the germ-free mouse placenta by altering the mRNA levels of well-known placental hormones, such as prolactins and PSG family members, as well as more than 400 newly identified proteins, some of which correlate with fetal weight and fetal glycemia. Moreover, we observed a reduction in the presence of fetal resorptions within the litter, suggesting a potential protective role of *B. breve* in pregnancy maintenance, potentially via hormone production, although further work is required to validate this hypothesis.

Maternal gut *B. breve* modulates placental physiology, as evidenced by the upregulation of nutrient transporters such as *Slc7a8* (large neutral amino acids) and *Slc16a4* (monocarboxylates), along with elevated levels of lactate and taurine in the junctional zone. These metabolic changes may reflect an enhanced capacity to meet the metabolic and biosynthetic demands of endocrine cells within this placental compartment. Additionally, maternal gut *B. breve* exerts its effect in a tissue-specific manner, as the abundance of certain nutrient carriers/transporters (e.g. *Slc2a1* and *Slc2a3*) and the altered metabolites in the placental endocrine zone differ from those in the placental transport zone or the fetal brain [19, 20].

*Bifidobacterium*, via degradation of complex carbohydrates, is a major producer of lactate [52]. This metabolite, which is required for a variety of cellular events, including energy regulation, redox signalling or hormone production in reproductive tissues [53], is transported via monocarboxylate transporters (MCTs – *Slc16* family) [54]. In our previous work, we found that *B. breve* can increase the expression of MCT transporters in the fetal brain [20]. Interestingly, in this new work, we have also observed elevated mRNA levels of *Slc16a4* and a trend for increased *Slc16a1* (*p = 0.06*) in response to *B. breve* administration. However, despite this increase at the mRNA level, protein levels of SLC16A1 in the BIF group were reduced (Table S4), suggesting a potential change in translational efficiency or post-translational regulation. Alternatively, shifts in the transmembrane lactate gradient could have influenced SLC16A1 stability or localization, leading to a potential reduction in the detectable levels of the protein, although further work is needed to explore this possibility [54]. Moreover, another limitation of the current study is the inability to distinguish between host- and *Bifidobacterium*-derived lactate. Future work should address this by employing specialized mass spectrometry approaches or administering ^13 C-labelled lactate to pregnant germ-free mice to trace its metabolic fate and determine whether the observed lactate increase originates from host metabolism or microbial production.

Another metabolite that was elevated in the placental endocrine tissue in response to *B. breve* supplementation was taurine, an important amino acid required for fetal organ development and cellular renewal of the placental syncytiotrophoblast layer (area of maternal-fetal exchange and hormone production) [55–57]. In fact, taurine is the most abundant amino acid in human placenta, indicating a crucial physiological role in this tissue [58]. Although the direct role of taurine or lactate in placental hormone production has not been tested, previous studies have shown that infusions of these metabolites can stimulate prolactin secretion [59, 60]. Future studies should validate these findings by administering taurine and/or lactate to pregnant mice and conducting in vitro experiments with placental cells/explants/organoids to assess changes in placental endocrine output.

Previous studies have shown that the increase in the level of *Bifidobacterium* during pregnancy is associated with pregnancy hormones like progesterone [61]. In the current study, we observed elevated levels of *Hmgcr*, a key enzyme involved in the *de novo* biosynthesis of fatty acids and cholesterol, which are essential for progesterone production [62]. Moreover, we found that the mRNA levels of certain forms of placental prolactins and PSGs, two important placental hormone families involved in a variety of functions including immune tolerance, angiogenesis and trophoblast invasion [63–66], were elevated in response to *B. breve* administration. Interestingly, recent work has found that *Hmgcr*, prolactins and PSGs can be modified by imprinted genes like *Igf2* or *H19* [26, 40, 41]. Although in our study we did not observe changes in the mRNA of *Igf2* and *H19* or protein abundance of IGF2, we observed that another member of the insulin-like growth factor family, IGF1R, was downregulated (Table S4). IGF1R plays a critical role in regulating placental function, by promoting cellular proliferation, angiogenesis, and metabolism [67, 68]. Moreover, IGF1R has been implicated in the pathogenesis of fetal growth restriction, with elevated protein levels observed in small babies, potentially as a compensatory mechanism to promote rapid growth [69]. Interestingly, although again we did not observe changes in the mRNA levels of the other paternally-expressed imprinted gene, *Peg3*, we found reduced levels of its protein (Table S4). Previous work has found that a loss of function of *Peg3* reduces the formation of the Jz, decreases expression of placental prolactins (*Prl8a1* and *Prl8a8*) and increases the expression of PSGs (*Psg17* and *Psg19*) [42]. While our current model does not allow us to directly attribute the observed changes in prolactin or PSG expression to reduced PEG3 protein levels, these findings raise the possibility of such a link. Future work using genetically modified models with reduced or ablated *Peg3* expression, combined with microbial interventions, could help determine whether *B. breve* has the capacity to rescue key aspects of placental endocrine function.

Additionally, we found that administering this bacterium not only rescued fetal growth and fetal glycaemia [19], but also reduced the occurrence of resorptions within the litter. These findings highlight a potential microbiota-mediated resilience mechanism that may help protect against pregnancy complications such as miscarriage or fetal growth restriction. Embryonic and fetal loss has been linked to altered hormone levels, including prolactins and PSGs [70, 71]. However, despite the observed increase in mRNA levels for most of these proteins, these elevations in mRNA levels did not translate into higher protein levels of placental prolactins or CD9 (notably, none of the PSGs evaluated were detected by LC-MS/MS). Taken together, these observations further suggest a mismatch between mRNA levels and protein production in the BIF group, indicating robust post-transcriptional regulation within the placenta. Such regulation may involve miRNAs, RNA-binding proteins, or altered protein turnover, all of which are known to be active during pregnancy [72–74]. Future studies employing approaches like translational profiling (e.g., Ribo-Seq) could help uncover the specific regulatory mechanisms underpinning these discrepancies. Moreover, it is important to note that our proteomic analysis was conducted at a single time point (GD16.5); thus, the effects of *B. breve* on the translational machinery and protein synthesis may vary over time. In fact, many of the proteins and pathways altered by *B. breve* supplementation suggest an impact on translation and protein synthesis. For instance, RL37, a ribosomal protein involved in translation [75], ICE2 and TSSK4, involved in transcription or gene regulation (Table S4), was among the top 10 downregulated proteins in response to *B. breve* supplementation. Proteomic analysis revealed downregulation of pathways related to RNA processing, translation, and the cell cycle, suggesting a suppression of core cellular functions. This may reflect a strategy to limit placental cell turnover, potentially contributing to the preservation of endocrine stability within the junctional zone. Conversely, we observed upregulation of pathways such as NF-κB signalling and polyamine metabolism, which are associated with immunometabolic reprogramming [76–78]. These changes may support enhanced placental function and sustained hormone production, aligning with the observed endocrine-specific adaptations in response to *B. breve* colonisation. Future studies should conduct a pregnancy time-course evaluation of both placental and maternal plasma to detect alterations in production and in the circulating levels of the aforementioned proteins, particularly in prolactins and PSGs.

Nonetheless, in this study we have identified over 400 proteins in the Jz influenced by maternal *B. breve* supplementation, with more proteins being downregulated than upregulated. In line with this, our previous RNA-seq analysis of the fetal liver between GF and BIF group showed that the number of downregulated genes (508) exceeded the upregulated genes (94) in the BIF group [19]. One possible explanation is that BIF-treated fetuses were larger than those in the untreated GF group [19], suggesting that the placenta may adapt by reducing certain endocrine or metabolic outputs once fetal growth has already been promoted, reflecting a feedback or homeostatic mechanism. Future work should validate this hypothesis by collecting samples at multiple time points of gestation. Among the different proteins altered by the maternal gut *Bifidobacterium*, some of them implicated in the regulation of metabolic processes during pregnancy and linked to certain pregnancy complications. For example, FETUB levels were increased in response to *B. breve* administration and is involved in lipid metabolism [79]. PKM2, elevated in the BIF group, is a metabolic enzyme involved in glycolysis that participates in trophoblast cell invasion and elevated in situations of fetal growth restriction [80]. Others like LUM (known as Lumican), also elevated in the BIF group, have been reported to be reduced in situations of preeclampsia causing defects in placental cell proliferation [81]. Additionally, a key strength of our study is the identification of 736 placental proteins that correlate with fetal growth, fetal glucose levels, and placental weight. Among these, we found that 29 placental Jz proteins were specifically regulated by maternal gut *B. breve*. For instance, HDAC1 or PPARG, which have been also linked to pregnancy complications [82, 83], correlates negatively with fetal weight and their abundance in the Jz is significantly reduced by *B. breve* administration. Conversely, proteins such as CFD and F13A1, which are altered in preeclampsia and spontaneous abortions, respectively [84, 85], correlated positively with fetal weight and were elevated following *B. breve* administration. Our correlation analysis not only highlight potential pathways through which *B. breve* may influence placental function, but also offers a basis for further exploration into less well-characterized aspects of placental endocrinology. By linking protein expression to key fetal outcomes, these findings may help identify candidate biomarkers and proteins of interest that could be involved in the development of fetal complications such as growth restriction or hypoglycemia. Further studies will be needed to validate these associations and to clarify their mechanistic significance.

### Limitations of the study

Our study has several limitations. As we only examined a single gestational time point, longitudinal studies, starting at the onset of gut colonization with *B. breve* and continuing throughout gestation will be necessary to better understand how placental endocrine function is regulated by *B. breve*. Moreover, further validation using maternal and fetal plasma or amniotic fluid is required to assess the magnitude of changes induced in the junctional zone. Future in vitro experiments using models, such as organoids or placental explants, could also provide insight into the biological mechanisms underlying changes in hormone production. However, it must be noted that these approaches may not fully recapitulate the complexity of the* in vivo* situation. Finally, a higher sample size and the inclusion of specific-pathogen-free (SPF) mice in the study, as in our previous work on the placental transport region [19], would have offered additional insight into interpreting the endocrine and metabolic changes observed in the junctional zone of the BIF group. In particular, SPF groups supplemented with or without *B. breve* would have helped clarify microbiota-dependent effects from those associated with the germ-free background.

### Summary

This study provides the first clear evidence that maternal gut *Bifidobacterium* significantly influences placental endocrine function. Although our work was conducted exclusively in GF mice and requires validation in additional animal models and, ultimately, in pregnant women, the findings may have societal relevance given the rising global consumption of probiotics, with approximately 14% of pregnant women taking these supplements [86]. Our results highlight the critical role of *Bifidobacterium* during pregnancy in regulating placental endocrine function. Although we did not include SPF mice to assess whether *B. breve* could restore altered protein levels to normal values, the study underscores the importance of monitoring pregnant women with gut microbiota perturbations.

In conclusion, our study demonstrates that the maternal gut microbiota regulates placental endocrine function by altering specific metabolites, nutrient carrier proteins, hormones, and the proteome in germ-free mice. These findings, beyond representing a potential adaptive microbial strategy to optimise fetal growth, offer key insights into fetal growth regulation and uncover novel proteins involved in feto-placental metabolism. Moreover, these proteins may act not only locally within the junctional zone but also exert paracrine effects, potentially contributing to the changes observed in the placental labyrinth zone and influencing fetal development [19, 20]. Together, the differentially regulated proteins and pathways identified may affect critical processes such as immune tolerance, angiogenesis, and antioxidant defence within the placental niche, with broader implications for fetal homeostasis and pregnancy health. Overall, our results highlight a broader role for the maternal gut microbiota in shaping feto-placental development and support the emerging concept of a gut-placental axis, paving the way for future mechanistic and translational studies.

## Supplementary Information

Below is the link to the electronic supplementary material.

Supplementary Material 1: Table S1. Fetal sex distribution for the samples used for gene expression analysis (qPCR), metabolomics and proteomics.

Supplementary Material 2: Table S2. List of primers used for qPCR.

Supplementary Material 3: Table S3. Maternal gut *B. breve* supplementation induces changes in placental endocrine zone metabolites. Data were analysed by one-way ANOVA, with group as fixed effect, and mean comparisons performed by Fisher’s test (general linear model, GLM). Litter size was included as a covariate. Data are displayed as mean ± SEM and as raw data. Statistical significance was set at *P* < 0.05.

Supplementary Material 4: Table S4. Maternal gut *B. breve* supplementation modifies the endocrine capacity of the placenta. List of proteins detected by LC-MS/MS, g:Profiler terms that are upregulated or downregulated, and correlation analysis between all proteins detected by LC-MS/MS, fetal weight, fetal glycaemia and placental weight (related to Fig. 4).

Supplementary Material 5: Figure S1. Gut colonization levels of *B. breve* determined in maternal faecal samples on gestational day (GD), 12 and 14. The original figure was published in our previous publication [19]. Figure S2. Effects of *B. breve* on the expression of *Igf2*, *H19*, and *Peg3* genes, as determined by qPCR. Data are presented as means ± SEM, with individual datapoints shown.

## Data Availability

The datasets used and/or analysed during the current study are available from the corresponding author on reasonable request.
