# Supplementary material for: Placental endocrine function is controlled by maternal gut Bifidobacterium in germ-free mice
Source: J Transl Med. 2025 Oct 7;23:1031. doi: 10.1186/s12967-025-07198-4 (PMC12502190; doi:10.1186/s12967-025-07198-4)
Supplement: Supplementary file 1 — Supplementary Material 1: Table S1. Fetal sex distribution for the samples used for gene expression analysis (qPCR), metabolomics and proteomics. [file 12967_2025_7198_MOESM1_ESM.docx]

**Table S1.** **Fetal sex distribution for the samples used for gene expression analysis (qPCR), metabolomics and proteomics.**

| **Technique** | **GF fetuses** | **BIF fetuses** |
| --- | --- | --- |
| qPCR | 7 (3 females and 4 males) | 9 (4 females and 5 males) |
| Metabolomics | 4 (2 females and 2 males) | 6 (3 females and 3 males) |
| Proteomics | 4 (2 females and 2 males) | 5 (2 females and 3 males) |
