# Supplementary material for: Placental endocrine function is controlled by maternal gut Bifidobacterium in germ-free mice
Source: J Transl Med. 2025 Oct 7;23:1031. doi: 10.1186/s12967-025-07198-4 (PMC12502190; doi:10.1186/s12967-025-07198-4)
Supplement: Supplementary file 2 — Supplementary Material 2: Table S2. List of primers used for qPCR. [file 12967_2025_7198_MOESM2_ESM.docx]

| **Gene ID** | **Forward (5'→3')** | **Reverse (5'→3')** |
| --- | --- | --- |
| *Slc2a1* | GCTTATGGGCTTCTCCAAACT | GGTGACACCTCTCCCACATAC |
| *Slc2a3* | GA TCGGCTCTTTCCAGTTTG | CAA TCA TGCCACCAACAGAG |
| *Slc7a8* | CCAGTGTGTTGGCCATGATC | TGCAACCGTTACCCCATAGAA |
| *Slc16a1* | GCCGTCCAGTAATGATCGCT | GCAAGCCCAAGACCTCCAAT |
| *Slc16a2* | GCTTCGGCTGGATAGTGGTG | CTCCGACCCATGCTGCTT |
| *Slc16a4* | GGCTGGCGGTAACAGAGTA | CGGCCTCGGACCTGAGTATT |
| *Tpbpa* | TGGTGTACGGTCAGTAAATTCG | CTGTACCCCCTCGAAACTGAC |
| *Igf2* | CTTGTTGACACGCTTCAGTTTG | GGGTGGCACAGTATGTCTCC |
| *H19* | CATTCTAGGCTGGGGTCAAA | GCCCTTCTTTTCCATTCTCC |
| *Peg3* | AGCACATCCCACTGTACGAA | TCTGCATTTGACCCCTGGAT |
| *Pcdh12* | GCCTGGTTAGGCTCTCTGTG | TTATTTCCTCGGTGGTTTGG |
| *Hmgcr* | CACGCTCATAGTCGCTGGAT | CACGACGGGAGACGTGATAG |
| *Stard1* | TCCTCGCTACGTTCAAGCTG | CGTCGAACTTGACCCATCCA |
| *Hsd3b1* | GTCATTCCCAGGCAGACCAT | CTGTTCCTCGTGGCCATTCA |
| *Cyp11a1* | GTCGAGATCCGGGCTTCTTT | GTCATCTCCAGCTCCGCAAT |
| *Cyp17a1* | TGGAGGCCACTATCCGAGAA | CACATGTGTGTCCTTCGGGA |
| *Prl2c2* | TGCAATACTTCTTTCCTTCCAACTC | ATCAGGAGCCATGATTTTGGA |
| *Prl3a1* | TGGCTCAGTACATCTCAAACCT | TTGTTCAGTGCTTGCAGGAG |
| *Prl3b1* | GGCTGCTCTTCCACATGTACC | TCACTTGCAACAGCTCCTGG |
| *Prl7b1* | CAGCACATCAATAGCCTTGC | TTGGTGATTTGAGTGGCAAA |
| *Prl8a8* | TCAGAGCTGCATCTCACTGC | GGGACATCTTTCATGGCACT |
| *Flt1* | AAAGGCTGAGCATCACTCCC | GGACTCCCTGCATCACTAACAA |
| *Psg17* | CAGGTGTACTCCTCTCTTTTCATCT | TGCTCCTTTGTACCAGGTAAGT |
| *Psg18* | ACACCCTACGAACTCTGACTC | TGTCACAGCACAAGGAATGG |
| *Psg19* | GGACATCGGATTCTACACCCT | GGACAGAGTTGAAAGCGTCA |
| *Psg21* | ACGTCCACATTTCTTCAGGTC | CGTCCTCCTTCAGCAACTCT |
| *Cd9* | GCTGGGATTGTTCTTCGGGT | GGGTTCATCCTTGCTCCGTA |
| *Actb* | GGCTGTATTCCCCTCCATCG | CCAGTTGGTAACAATGCCATGT |
| *Krt18* | CAAGACCTGAACCGTCGCCT | ATTCGCAAAGATCTGAGCCCT |

**Table S2. List of primers used for qPCR**
