# Supplementary material for: Placental endocrine function is controlled by maternal gut Bifidobacterium in germ-free mice
Source: J Transl Med. 2025 Oct 7;23:1031. doi: 10.1186/s12967-025-07198-4 (PMC12502190; doi:10.1186/s12967-025-07198-4)

Figure S1. Gut colonization levels of B. breve determined in maternal faecal samples on gestational day (GD), 12 and 14. The original figure was published in our previous publication [19].


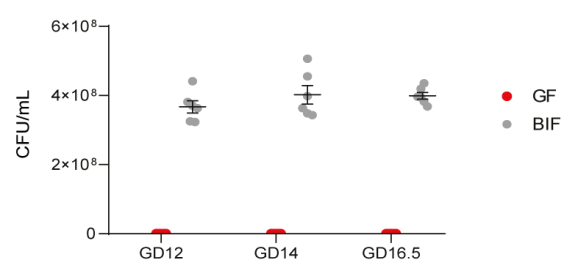


Figure S2. Effects of *B. breve* on the expression of *Igf2*, *H19*, and *Peg3* genes, as determined by qPCR. Data are presented as means ± SEM, with individual datapoints shown.


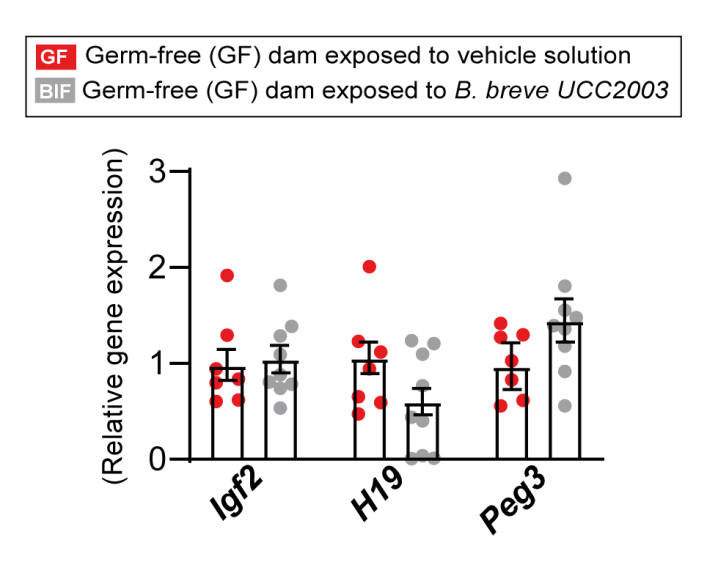

Supplement: Supplementary file 5 — Supplementary Material 5: Figure S1. Gut colonization levels of B. breve determined in maternal faecal samples on gestational day (GD), 12 and 14. The original figure was published in our previous publication [19]. Figure S2. Effects of B. breve on the expression of Igf2, H19, and Peg3 genes, as determined by qPCR. Data are presented as means ± SEM, with individual datapoints shown. [file 12967_2025_7198_MOESM5_ESM.docx]
